# A Newly Established Cuproptosis-Associated Long Non-Coding RNA Signature for Predicting Prognosis and Indicating Immune Microenvironment Features in Soft Tissue Sarcoma

**DOI:** 10.1155/2022/8489387

**Published:** 2022-07-06

**Authors:** Jun Han, Yunxiang Hu, Sanmao Liu, Jian Jiang, Hong Wang

**Affiliations:** ^1^Department of Orthopedics, Dalian Municipal Central Hospital Affiliated of Dalian Medical University, No. 826, Southwestern Road, Shahekou District, Dalian, Liaoning Province 116021, China; ^2^Dalian Medical University, No. 9, West Section of South Lvshun Road, Dalian, Liaoning Province 116044, China; ^3^The First Affiliated Hospital of Dalian Medical University, Dalian, Liaoning Province, China

## Abstract

Cuproptosis, a new type of programmed cell death, is involved in the development and progression of malignancies. The study of cuproptosis-associated long non-coding RNAs (lncRNAs) in soft tissue sarcomas (STSs) is however limited. There is also uncertainty regarding the prognostic accuracy of cuproptosis-associated lncRNAs in STSs and their relationship to the tumor immune microenvironment. The aim of this study was to determine the prognostic significance of cuprotosis-associated lncRNAs in STSs and their relationship to the tumor immune microenvironment. Transcriptomic and clinical data from patients with STSs were obtained through The Cancer Genome Atlas (TCGA). Overall, 259 patients were randomly allocated to a training group or a testing group. In the training group, a cuproptosis-associated lncRNA signature was constructed, and the signature was verified in the testing group. On the basis of risk scores and clinical features, we later developed a hybrid nomogram. We also performed functional and tumor immune microenvironment analysis based on the cuproptosis-associated lncRNA signature. A signature of 5 cuproptosis-associated lncRNAs was created. Based on this signature, we categorized STS patients into high-risk and low-risk groups. The study showed that patients at high risk had a worse prognosis than those at low risk. A nomogram was then constructed combining clinical characteristics with the risk scores, and it was shown to have credible predictive power. Functional enrichment and tumor immune microenvironmental analyses showed that high-risk STSs tend to be immunologically sensitive tumors. In our study, we found a cuproptosis-associated lncRNAs signature, which serves as an independent prognostic indicator. Cuproptosis-associated lncRNAs may play a role in the tumor immune microenvironment, which might be a therapeutic target for patients with STSs.

## 1. Introduction

Sarcomas are solid tumors originating from the mesenchymal tissue and are classified as osteosarcoma and STSs [1], with more than 50 histological types. The incidence of human osteosarcoma and STS is 1 per 100,000 and (4-5)/100,000 per year, respectively, accounting for approximately 1% of all malignancies [2]. Despite the fact that STSs can arise anywhere in the body, extremities account for 60–70% [3]. The mechanisms of biological behavior of human STSs, such as occurrence, proliferation, metastasis, resistance to radiotherapy, and recurrence, need to be studied more thoroughly from various aspects. lncRNAs are RNA molecules with transcripts that are longer than 200 nucleotides, which were initially thought to be by-products of RNA polymerase II transcription [4]. lncRNAs are important regulatory molecules in the process of tumorigenesis [5–7]. In addition, lncRNAs are also associated with tumor invasion, infiltration, metastasis, and prognosis [8]. Currently, due to the advancement of high-throughput sequencing technology, more and more functions of lncRNAs are being annotated. lncRNAs such as T⁃ALL⁃R⁃LncR1 and MEG3 have been found to be associated with rhabdomyosarcoma [9, 10], and it has been shown that polyadenylated nuclear noncoding RNA (PAN RNA) is associated with Kaposi's sarcoma [11]. However, the function of many other lncRNAs in STSs remains unknown.

Depending on the mechanism of cell death, there are different ways of cell death, and the common ones are apoptosis, necroptosis, pyroptosis, and ferroptosis [12]. Similar to ferroptosis, copper is an indispensable trace element in all living organisms and is usually maintained at very low levels in mammalian cells. Intracellular concentrations of copper ions that exceed the threshold for maintaining homeostatic mechanisms likewise exhibit cytotoxicity. Tsvetkov et al. [13] first found that cuproptosis occurs through the direct binding of copper to the lipidated components of the tricarboxylic acid cycle (TCA). This led to lipid-acylated protein aggregation, and loss of iron-sulfur cluster proteins, which in turn triggered proteotoxic stress and ultimately cell death. However, studies of cuproptosis-associated lncRNAs in STSs are limited. Specifically, there is uncertainty regarding the prognostic accuracy of curproptosis-associated lncRNAs and their relationship to tumor immune microenvironment in STSs. Therefore, the aim of our study was to identify cuproptosis-associated lncRNAs in STSs, as well as to understand the role of cuproptosis-associated lncRNAs in tumor immune microenvironment and prognosis, which not only sheds light on the signaling pathways and molecular mechanisms involved in the cuproptosis action in STSs, but also might provide new perceptions for patients with STSs seeking immunotherapy.

## 2. Materials and Methods

### 2.1. Data Retrieval and Identification of Cuproptosis-Associated lncRNAs

We downloaded transcriptomic and clinical data from the TCGA database of a total of 261 patients with STSs (Table S1). Copy number variation (CNV) data and somatic mutation data (level (2)) for STS cases have also been downloaded from the TCGA data portal (https://tcga-data.nci.nih.gov/tcga/dataAccessMatrix.htm). Study participants with incomplete clinical information were excluded. Cuproptosis-associated genes were obtained from a literature search [13–17]. We then assessed the correlation of cuproptosis-associated lncRNAs with cuproptosis-associated genes by Pearson's correlation analysis. In order to identify cuproptosis-associated lncRNAs, Pearson's correlation coefficients higher than 0.2 (*R* > 0.2) and *P* values less than 0.05 (*P*^*∗*^0.05) were required.

### 2.2. Construction and Verification of a Cuproptosis-Associated lncRNA Signature

**Figure 1:**
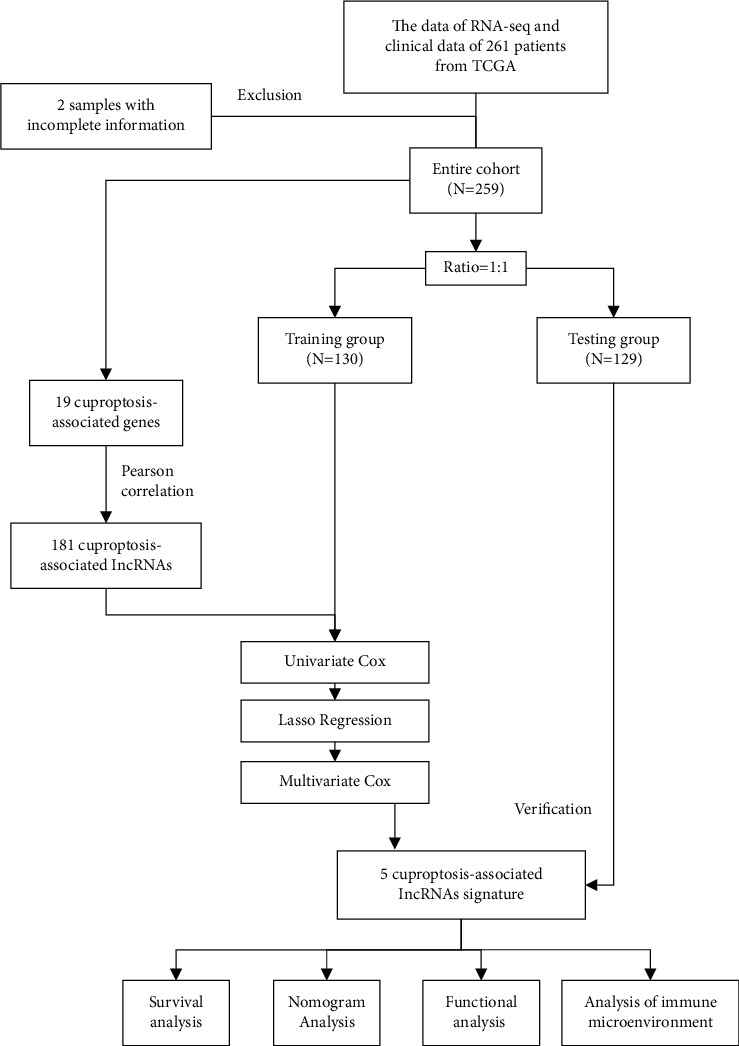
Flowchart of the study.

We included 259 STS patients who were randomly assigned to a training or testing group (Figure 1). In the training group, by combining the analysis of univariate Cox regression, LASSO Cox regression, as well as multivariate Cox regression, a cuproptosis-associated lncRNAs signature was constructed. Finally, a risk score for each individual was computed using this prognostic signature. The risk score was calculated using the following formula: risk score = (normalized expression level of each cuproptosis-associated lncRNA *∗* corresponding correlation coefficient). Based on the median value of the risk score, the training group, testing group, and all patients were classified into high-risk and low-risk groups, respectively. Overall survival (OS) was compared between the high-risk and low-risk groups in the training group, testing group, and all patients respectively by Kaplan–Meier analysis.

### 2.3. Construction of a Predictive Nomogram

We later created a hybrid nomogram using the “rms” *R* package that incorporates the lncRNA signature and clinicopathological features of STS patients to predict their OS (1-, 3-, and 5-year). For determining the predictive power of a nomogram, calibration curves and consistency indices (C-index) were used.

### 2.4. Function Enrichment Analysis

Based on the risk scores, all samples were categorized into high-risk and low-risk groups. The “clusterProfiler” package in *R* was used to analyze enrichment analyses of Gene Ontology (GO), FDR < 0.05, and Kyoto Encyclopedia of Genes and Genomes (KEGG), FDR < 0.01, between the high-risk and low-risk groups.

### 2.5. Analysis of Tumor Immune Microenvironment between the Patients in High-Risk and Low-Risk Groups

We calculated the ratio of each tumor-infiltrating immune cell in all patients using the CIBERSORT algorithm [18]. Results produced by CIBERSORT were filtered at 0.05 *P* value. To evaluate the difference of the tumor immune microenvironment between the high-risk group and low-risk group, immune functions and each immune cell were compared between the high and low-risk groups. Tumor mutation burden (TMB) [19] and tumor immune dysfunction and exclusion (TIDE) [20] were also analyzed in the patients in predicting their reactions to immunotherapy.

### 2.6. Statistical Analysis

R was used to conduct all statistical analyses (v4.0.5). If the statistical significance level was not specifically indicated, it was assumed to be *P* < 0.05.

## 3. Results

**Figure 2:**
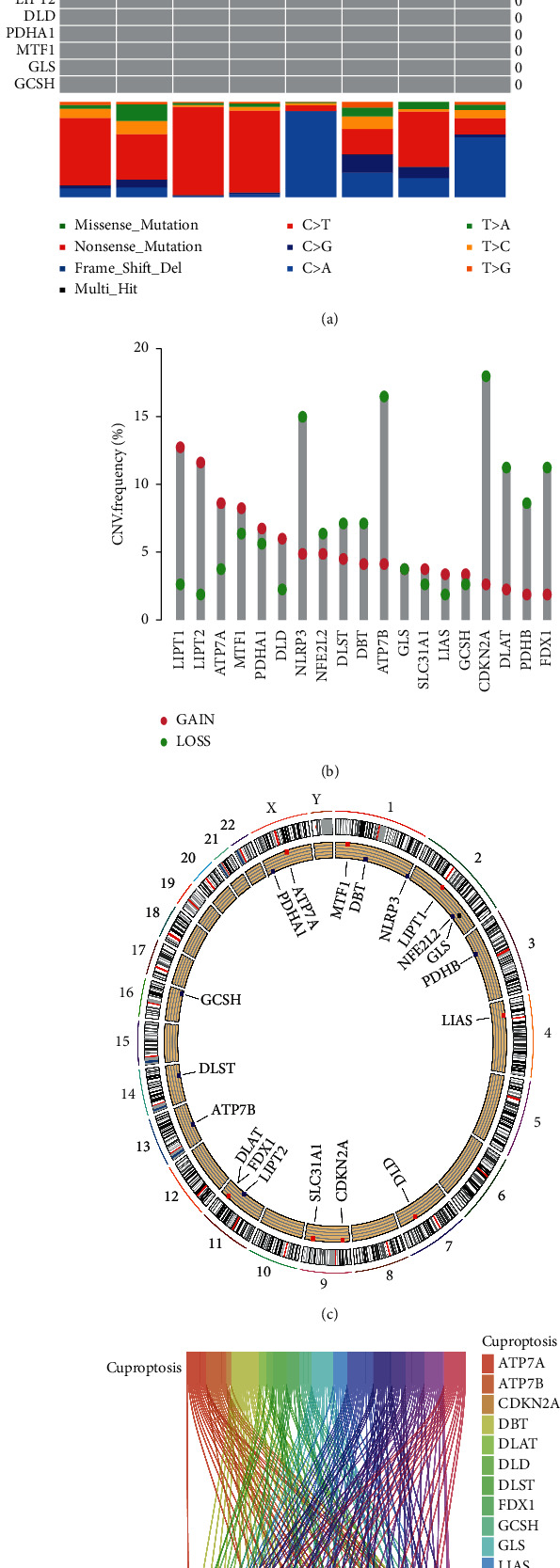
Mutation changes and expression changes of cuproptosis-associated genes in the patients. (a) In each waterfall plot, mutation information is presented for each gene associated with cuproptosis, and each mutation type is indicated by the color at the bottom. In the abovementioned bar chart, the numbers on the left represent the mutation burden whereas on the right are the mutation frequencies. (b) Frequencies of CNV, gain, and loss among cuproptosis-associated genes. (c) Position of altered CNV in cuproptosis-associated genes on 23 chromosomes. (d) 181 cuproptosis-associated lncRNAs correlated with 19 cuproptosis-associated genes.

Overview of mutation changes and expression changes of cuproptosis-associated genes in our patients: it is shown that 8 of 237 STS patients were with cuproptosis-associated genetic mutations (Figure 2(a)). CNV frequencies of cuproptosis-associated genes in our patients are shown in Figure 2(b). The position of altered CNV in cuproptosis-associated genes of our patients on their respective chromosomes is shown in Figure 2(c).

### 3.1. Construction of a Cuproptosis-Associated lncRNA Signature

**Figure 3:**
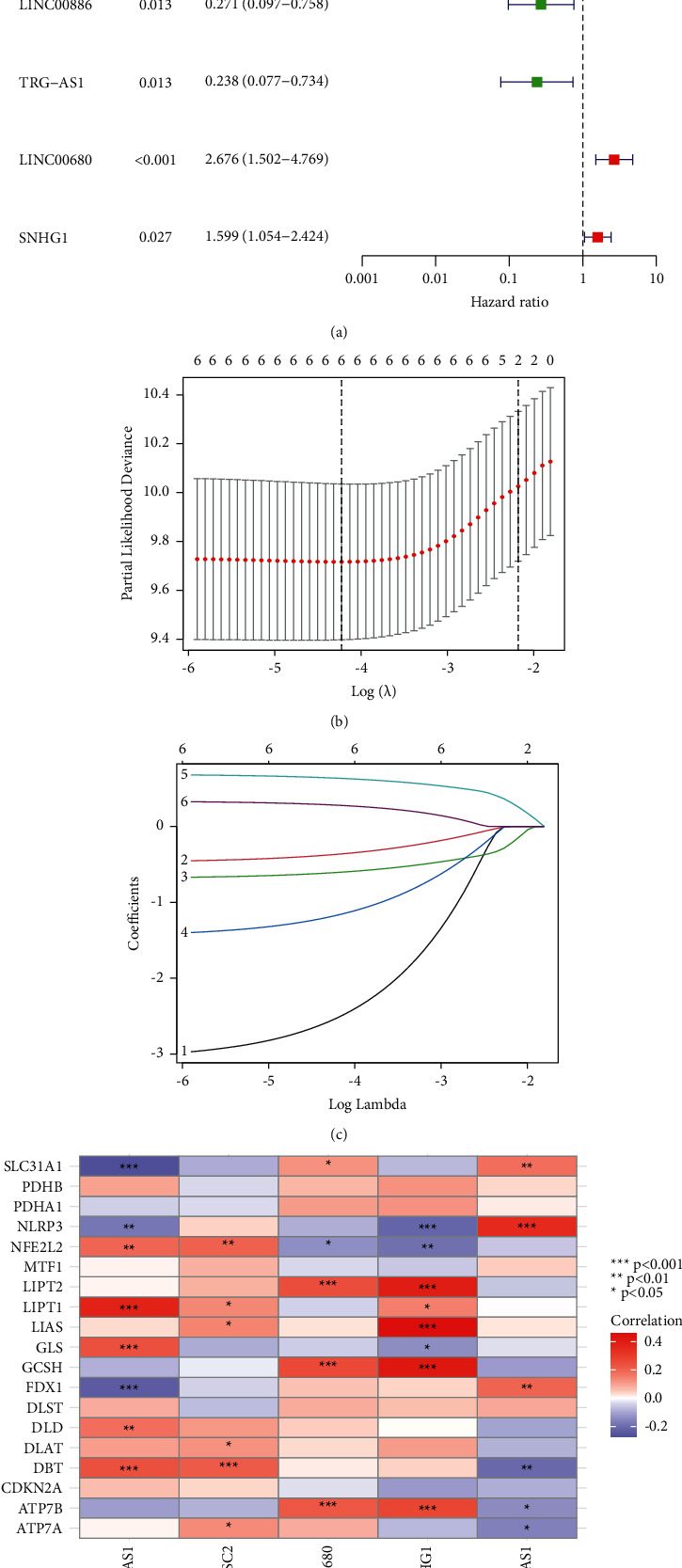
The construction of a prognostic signature in STS patients. (a) The univariate Cox regression analysis between cuproptosis-associated lncRNAs and OS of STSs is shown in the forest plots. The *P*-values were obtained by univariate cox regression. (b) According to minimum criteria, six cuproptosis-associated lncRNAs were selected by the least absolute shrinkage and selection operator (LASSO) regression model. (c) In LASSO regression, the coefficients of cuproptosis-associated lncRNAs were calculated. (d) The correlations between cuproptosis-associated genes and the 5 prognostic cuproptosis-associated lncRNAs in the proposed signature.

We enrolled 259 patients with STSs and these patients were randomly separated into the training group (*n* = 130) or the testing group (*n* = 129) in a ratio close to 1 : 1. The clinical data of all patients are specified in Table S1. It revealed no statistically significant difference in the clinical characteristics between the training group and the testing group. Following the obtainment of 19 cuproptosis-associated genes from the literature review, 181 cuproptosis-associated lncRNAs emerged with these genes by Pearson's correlation were calculated (Figure 2(d)). In our training group, first, in the univariate Cox regression analysis, we detected six cuproptosis-associated lncRNAs with a prognostic value (Figure 3(a)). Second, LASSO Cox regression was performed to reduce multicollinearity, which resulted in the selection of six lncRNAs (Figure 3(b)). Third, a subsequent multivariate analysis highlights 5 cuproptosis-associated lncRNAs (ADAMTS9 − AS1, CASC2, LINC00680, SNHG1, and TRG − AS1) for prognosis based on the lowest AIC (Figure 3(c)) [21]. Here, we explain how the risk score is computed based on the expression levels of each lncRNArisk score=ADAMTS9−AS1×0.624842897955339; CASC2×−3.58535963345813; LINC00680×0.631698088926909; SNHG1×0.384954590110563; TRG−AS1×−1.38127131637069.

The cuproptosis-associated genes and these 5 cuproptosis-associated lncRNAs were correlated. For example, ADAMTS9−AS1 was positively correlated with LIPT1, GLS, etc., and negatively with SLC31A1, FDX1, etc. SNHG1 was positively correlated with LIAS, GCSH, etc., and negatively with NLRP3, NFE2L2, etc. (Figure 3(d))

**Figure 4:**
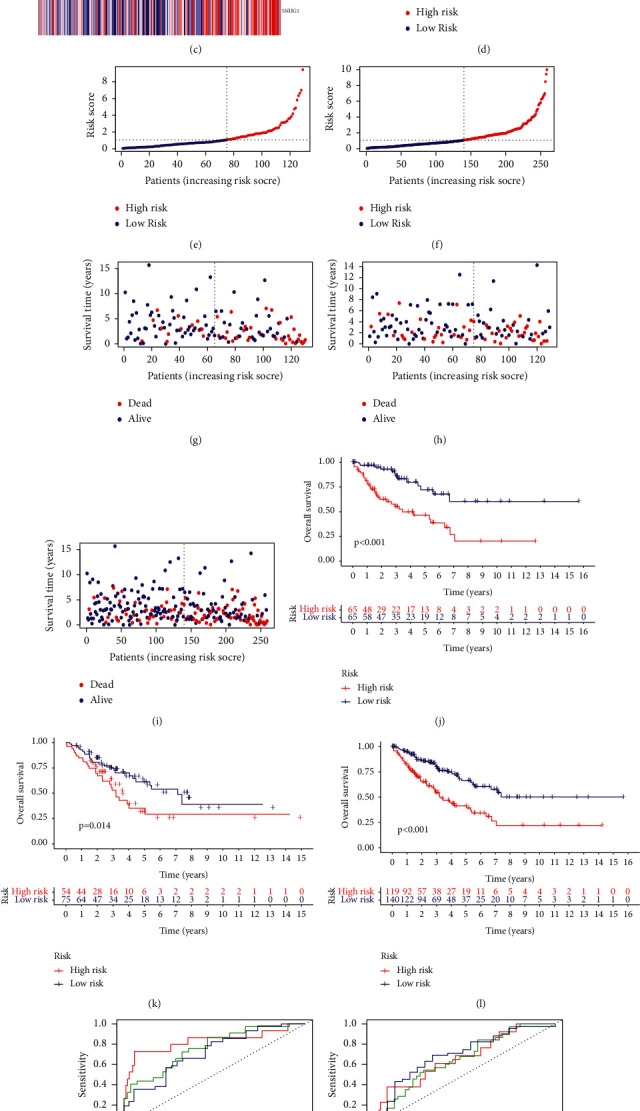
The prognostic performance of the 5 cuproptosis-associated lncRNAs of the signature in the training group, testing group, and all patients. (a–c) The expression heatmap of 5 cuproptosis-associated lncRNAs in the training group, testing group, and all patients. (d–f) The distribution of the risk scores in the training group, testing group, and all patients. (g–i) In the training group, testing group, and all patients, the scatter plots show whether the samples were alive or not. (j–l) The K-M curves depicting the OS in high-risk and low-risk groups in the training group, testing group, and all patients. (m–o) The prognostic accuracy of the risk scores in the training group, testing group, and all patients was verified by the ROC curve. ROC, receiver operating characteristic.

Patients in the training group were categorized into the high-risk group (*n* = 65) and low-risk (*n* = 65) group using the median risk score. Each patient's lncRNA expression levels, risk status, and survival outcome are demonstrated in Figures 4(a), 4(d), and 4(g). Kaplan–Meier analysis clearly demonstrated that the OS in the high-risk group was worse than that in the low-risk group (Figure 4(j)). The AUC was 0.831 at 1 year, 0.766 at 3 years, and 0.721 at 5 years (Figure 4(m)).

### 3.2. Verification of the Cuproptosis-Associated lncRNA Signature

To further verify the accuracy of the cuproptosis-associated lncRNAs signature, we calculated a risk score for each individual in the testing group using the same formula we used in the training group. The patients were then classified into a high-risk group (*n* = 54) and a low-risk group (*n* = 75) based on the same cut-off values as the training group. Each patient's lncRNA expression levels, risk status, and survival outcome are elucidated in Figures 4(b), 4(e), and 4(h). The results of Kaplan–Meier analysis demonstrated a relatively poor prognosis for STS patients in the high-risk group (Figure 4(k)). The AUC was 0.669 at 1 year, 0.658 at 3 years, and 0.699 at 5 years (Figure 4(n)).

Finally, similar to the training and testing groups, we obtained consistent results comparing all patients with the same cut-off values. Each patient's lncRNA expression levels, risk status, and survival outcome are depicted in Figures 4(c), 4(f), and 4(i). The results of Kaplan–Meier analysis demonstrated a relatively poor prognosis for STS patients in the high-risk group (Figure 4(l)). The AUC was 0.750 at 1 year, 0.701 at 3 years, and 0.669 at 5 years (Figure 4(o)).

**Figure 5:**
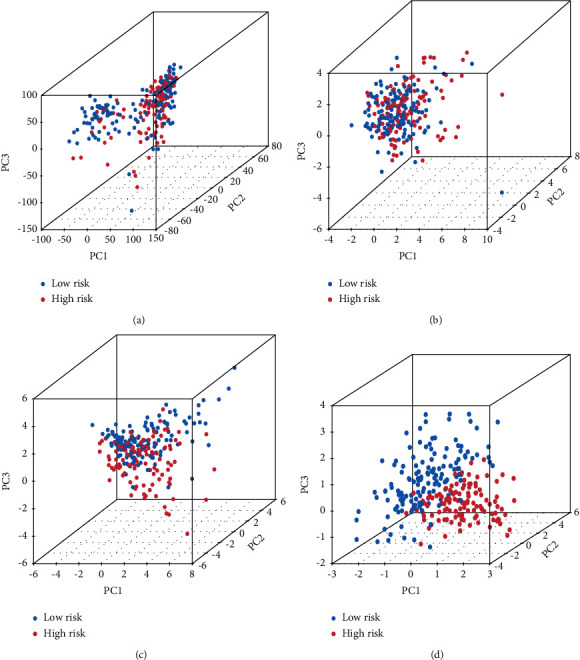
The results of principal component analysis. A comparison of gene expression levels in high- and low-risk patients, based on (a) the expression of all examined genes, (b) cuproptosis-associated genes, (c) cuproptosis-associated lncRNAs, and (d) the 5 lncRNAs of the prognostic signature.

On the basis of the entire examined genes (Figure 5(a)), 19 cuproptosis-associated genes (Figure 5(b)), 181 cuproptosis-associated lncRNAs (Figure 5(c)), and 5 cuproptosis-associated lncRNAs of the signature (Figure 5(d)), the difference between high-risk and low-risk individuals is ascertained using principal component analysis (PCA) via the “ggplot2” *R* package. The results showed that there is a relatively wide range of gene expression between high-risk and low-risk patients.

### 3.3. Construction of a Nomogram to Predict Patients' Survival

**Figure 6:**
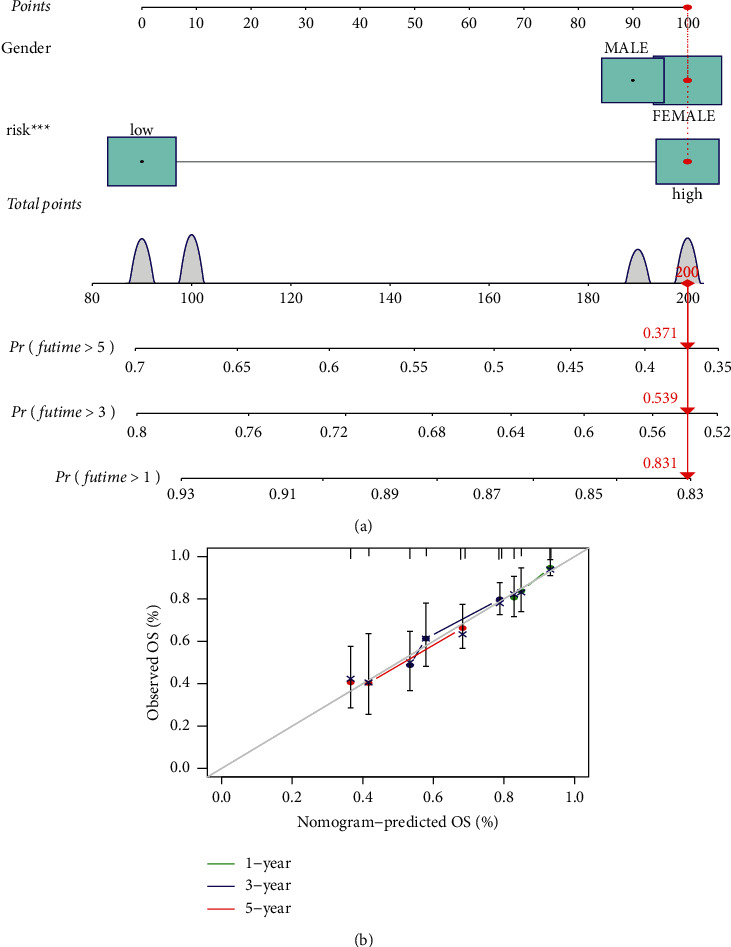
A hybrid nomogram based on cuproptosis-associated lncRNA signature for OS of STS patients. (a) A hybrid nomogram model to predict the 1-, 3-, and 5-year OS of STSs in all patients. (b) Calibration curves of the nomogram to predict the 1-, 3-, and 5-year OS of STSs in all patients.

Considering the inconvenient clinical utility of the cuproptosis-associated lncRNA risk score in predicting OS of patients with STSs, we later combined the cuproptosis-associated lncRNAs risk scores with clinicopathological features to create a hybrid nomogram model for predicting 1-, 3-, and 5-year OS (Figure 6(a)). Predictors included the risk score and gender. The subsequent calibration plots suggested that the proposed model performed similarly to an ideal model (Figure 6(b)).

### 3.4. Functional Enrichment Analysis

**Figure 7:**
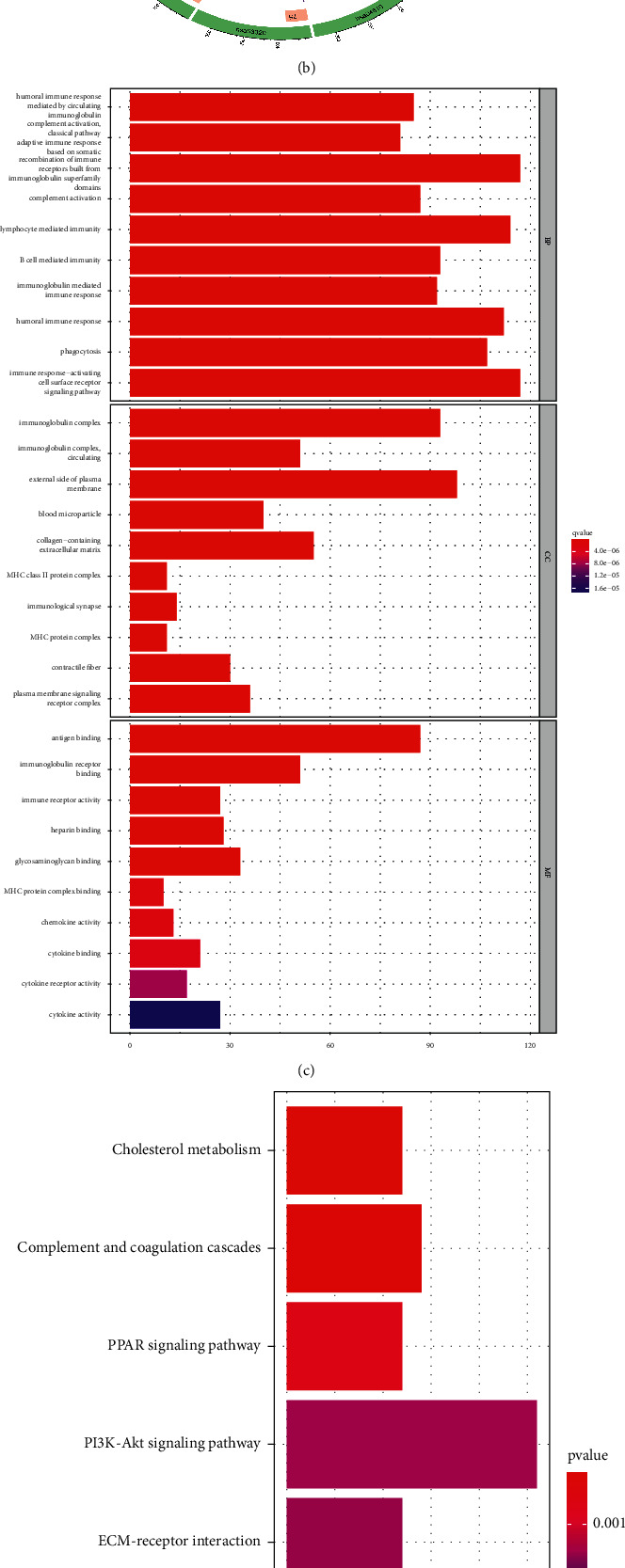
Analysis of GO enrichment and KEGG pathways. (a–c). The DEGs between patients with high risk and patients with low risk have been shown to be enriched mostly in immune-associated biological processes according to the GO enrichment analysis. (b–d) According to the KEGG pathway analysis, it is elucidated that the DEGs between high-risk and low-risk groups were typically enriched in complement and coagulation cascades, cholesterol metabolism, and PPAR signaling, etc.

In the high- and low-risk groups, we analyzed differentially expressed genes (DEGs) using GO enrichment and KEGG pathway analyses. Results of GO showed that DEGs in the high-risk and low-risk groups were enriched primarily in biological processes relating to immunity, such as humoral immunity, lymphocyte-mediated immunity, and adaptive immunity (Figures 7(a), 7(c)). The results of KEGG pathway analyses indicated that DEGs in the high-risk and low-risk groups tend to be enriched in many pathways such as cholesterol metabolism, PPAR signaling pathway, complement and coagulation cascades, as well as the PI3K-Akt signaling pathway. (Figures 7(b), 7(d)).

### 3.5. Tumor Immune Microenvironment of Soft Tissue Sarcoma between High-Risk and Low-Risk Patients

**Figure 8:**
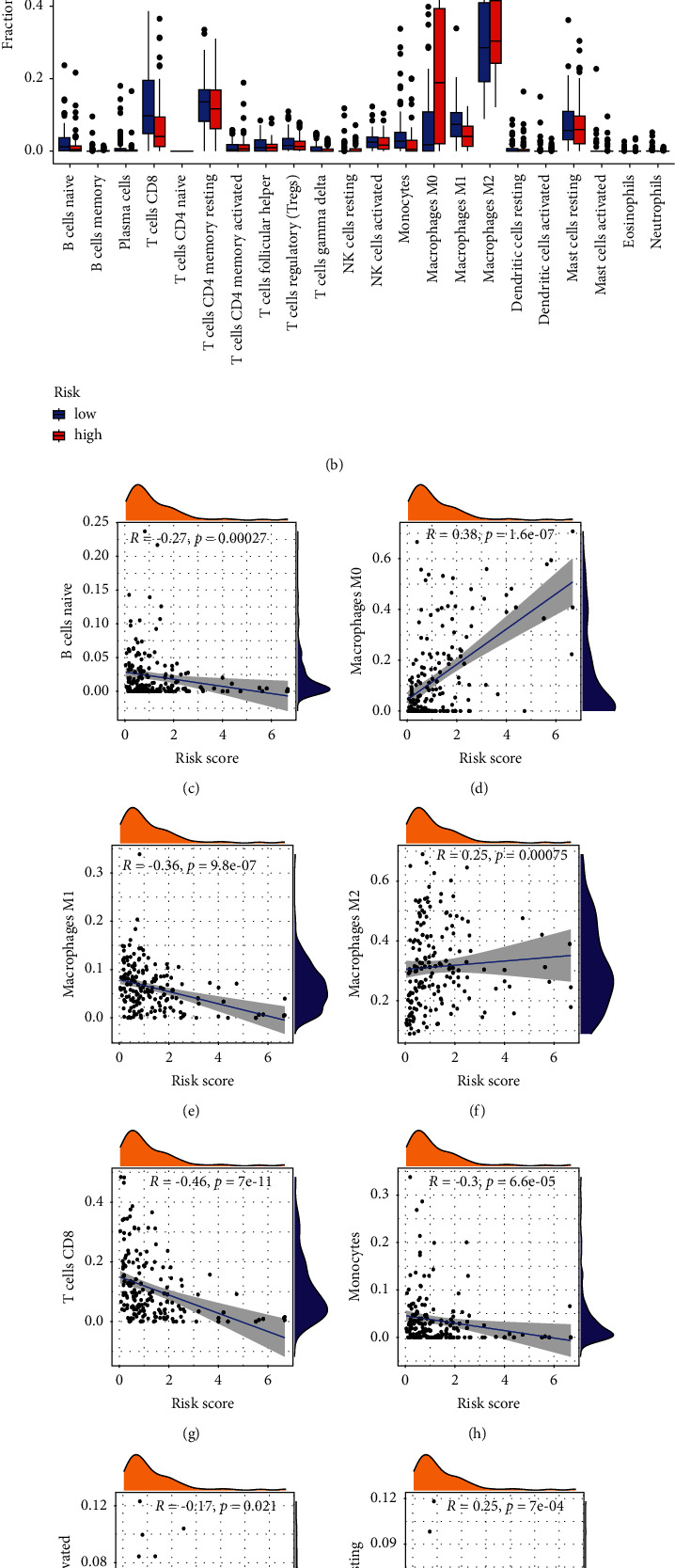
Comparison of immune infiltrating cells between high- and low-risk groups. (a) The proportion of immune cell types in all patients between high- and low-risk groups. (b) The proportion of 22 infiltrating immune cell types between the high-risk group and low-risk group. (c–i) Correlations between the abundance of 22 infiltrating immune cells and 5 lncRNAs in the proposed signature.

We calculated the proportion of various tumor-infiltrating immune cells using the CIBERSORT algorithm to explore the relationship between the risk score and the tumor immune microenvironment in all patients. The results showed that tumor-infiltrating immune cells differed significantly between high-risk and low-risk individuals (Figures 8(a), 8(b)). As shown in Figure 8(b), patients in the high-risk group had significantly lower proportions of tumor-infiltrating B cells naive, plasma cells, T cells CD8, monocytes, and M1 macrophages. High-risk patients, however, had significantly more resting NK cells and M0 macrophages infiltrating the tumor. Based on the CIBERSORT algorithm, a correlation was found between the risk score and the abundance of immune cells (Figures 8(c)–8(i)). As can be seen in the scatter diagrams, the risk score was positively correlated with the number of M0 macrophages, M2 macrophages, and NK cells resting, and negatively correlated with naive B cells, M1 macrophages, monocytes, NK cells activated, plasma cells, T cells CD8, and follicular helper T cells.

**Figure 9:**
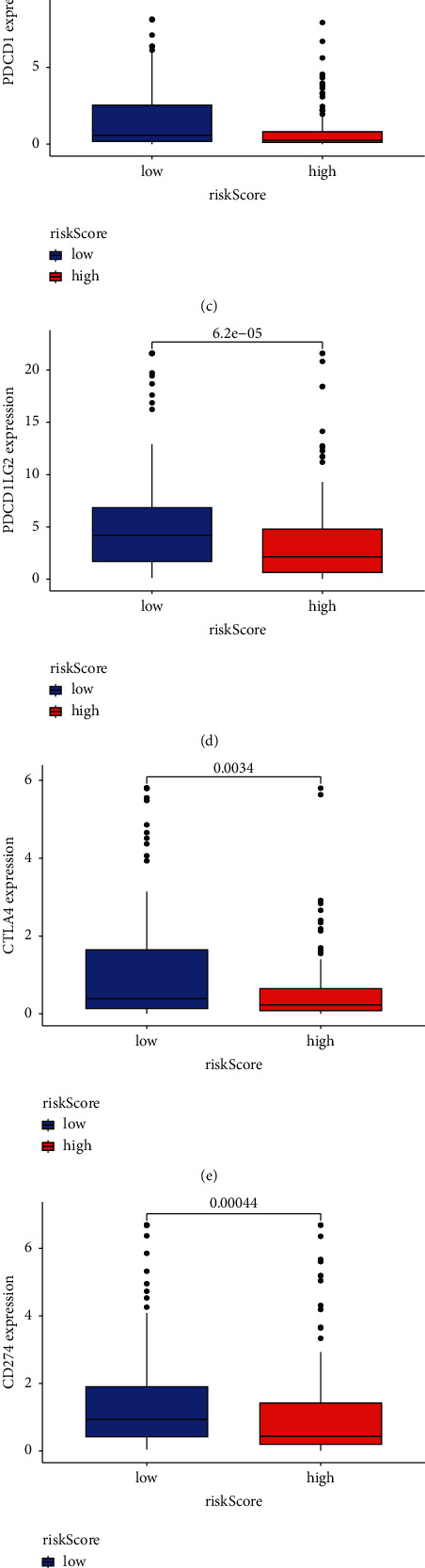
Comparison of immune-associated functions and immune checkpoint molecules between high- and low-risk groups. (a) The correlations between immune cells and the 5 lncRNAs. (b) GSVA of immune-associated functions between high-risk and low-risk groups. (c–h) The differences of immune check-point molecules between high-risk and low-risk groups.

In addition, we investigated the relationship between the 5 lncRNAs in the proposed signature and the number of immune cells. We noticed that the majority of immune cells exhibited a significant correlation with these 5 lncRNAs (Figure 9(a)). Immune-related functions such as Type_II_IFN_reponse, APC_co_stimulation, CCR, parainflammation, APC_co_inhibition, HLA, cytolytic_activity, check-point, T_cell_co-stimulation, inflammation-promoting, T_cell_co-inhibition, MHC_class_I, and Type_I_IFN_reponse were significantly more abundant in the low-risk group, according to a correlation analysis based on GSVA package ssGSEA (Figure 9(b)). We later compared the immune checkpoint molecules between the two groups, and we discovered that the low-risk group exhibited much higher levels of expression of PDCD1, PDCD1LG2, CTLA4, CD274, HAVCR2, and IDO1. (Figures 9(c)–9(h)).

**Figure 10:**
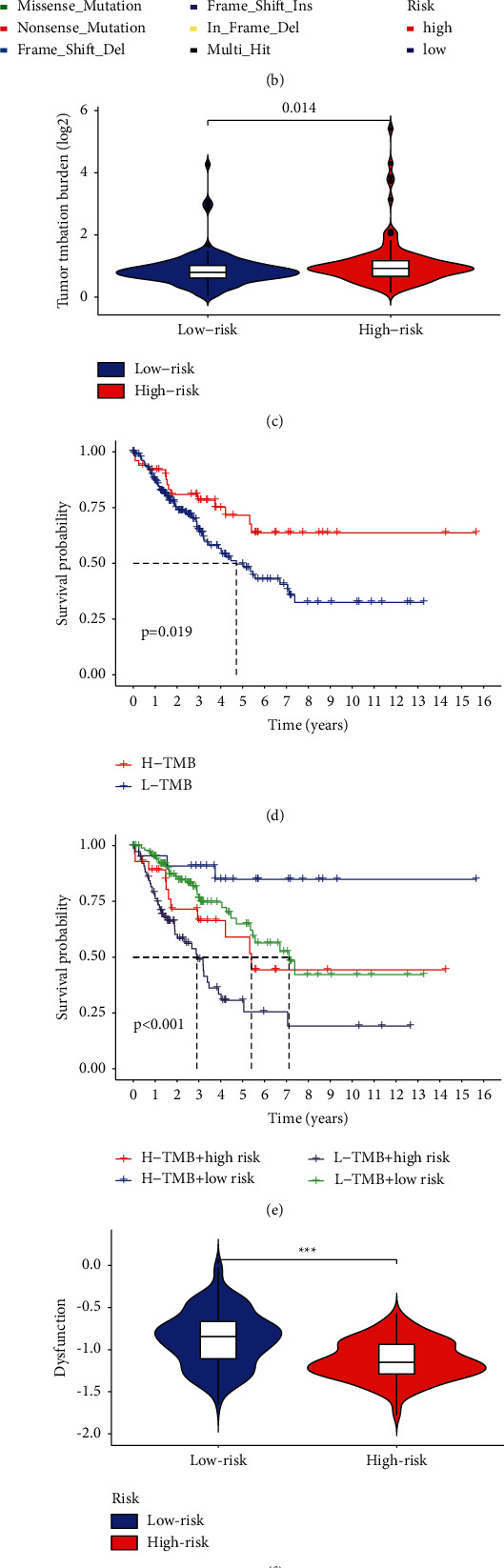
Comparison of mutation profiles, TMB, and TIDE in high-risk and low-risk groups. (a) The mutation profile of high-risk individuals. (b) The mutation profile of low-risk individuals. (c) Violin plot showing the difference of TMB between the high- and low-risk groups. (d) The K-M curves of H-TMB patients and L-TMB patients. (e) The K-M curves of H-TMB patients and L-TMB patients in the high-risk and low-risk groups. (f–h). Immunotherapy response between high- and low-risk groups. Violin plot illuminating the difference of the dysfunction score, exclusion score, and TIDE between the high-risk and low-risk groups.

Then, the changes in somatic mutation distribution between the high-risk and low-risk groups were examined; we noticed that the mutation rate was 74 (69.81%) of 106 in the high-risk groups and the top three mutated genes were TP53, ATRX, and TTN, while the mutation rate was 78 (60.47%) of 129 samples in the low-risk and the top tree mutated genes were TP53, ATRX, and RB. Notably, significantly more ATRX and TTN mutations were detected in patients with high risk than patients with low risk. However, a completely opposite trend was observed regarding TP53 mutation levels (Figures 10(a), 10(b)). TMB is an indicator associated with a better response to ICB treatment. The analysis of patients' mutation data showed that a higher TMB was found in the high-risk group compared to the low-risk group (Figure 10(c)), suggesting that the high-risk group might benefit from immunotherapy. Overall, survival was better for patients with a higher TMB (Figure 10(d)). We further compared the TMB in the high-risk and low-risk groups. It demonstrated that patients in the low-risk group with higher TMB had the best survival probability while patients in the high-risk group with lower TMB had the worst survival probability (Figure 10(e)). TIDE is a computational framework for modeling the two main mechanisms of tumor immune escape that can provide predictive results regarding immunotherapy. In order to better demonstrate the predictive power of risk scores for immunotherapy, we applied TIDE to our patients. Surprisingly, TIDE was negatively correlated with the risk scores (Figures 10(f)–10(h)).

## 4. Discussion

Despite significant improvements in the survival rate following aggressive multidisciplinary treatment encompassing surgery, radiotherapy, chemotherapy, and immunotherapy, the prognosis for patients with STSs remains poor [22, 23]. Even when patients share the same clinical risk factors, their prognosis and treatment outcomes may vary widely [24]. Therefore, the identification of effective therapeutic targets for the diagnosis and treatment of STSs is crucial. Recently, Tsvetkov et al. [13] identified cuproptosis as a novel type of programmed cell death with a dual function in tumor development and treatment; therefore, deciphering the biological process of cuproptosis in tumor cells might lead to new therapeutic targets. The large number of lncRNAs produced by human cells contributes an important role to various biological processes, including genome expression and cell differentiation [25]. Recent studies suggest that an abnormal expression of lncRNA may play a role in the progression and development of cancer [26, 27]. However, there is currently little research investigating cuproptosis-associated lncRNAs. Notably, this is the first comprehensive investigation of the role of cuproptosis-associated lncRNAs involved in the development of STSs.

Researchers in 2020 analyzed patients with high-grade STS samples, categorized by OS, and identified 7 genes such as CD36 andNCAM1 that are associated with a poor prognosis, and 6 genes such as BIRC5 and LAG3 that are associated with a good prognosis [28]. In our study5 lncRNAs were eventually identified as a cuproptosis-associated lncRNA signature. We found that the genes co-expressed with these 5-cuproptosis associated lncRNAs were NLRP3, LIPT2, LIPT1, LIAS, GLS, GCSH, DBT, and ATP7B, which were also correlated with the prognosis. As a result, we later developed a risk score dividing STS patients into the high- and low-risk groups, and our results demonstrated a significant difference in OS between the two groups. A further finding was that the risk scores could accurately predict patient prognosis without regard to traditional clinical risk markers or molecular factors. And then, a predictive nomogram was created by integrating the risk score with gender, thereby further improving its utility and making the risk score easier to use. For a better understanding of the relationship between cuproptosis-associated lncRNAs and STSs, functional enrichment analysis of GO and KEGG were undertaken. The results of GO showed that DEGs in the high-risk and low-risk groups were enriched primarily in biological processes relating to immunity, such as humoral immunity, lymphocyte-mediated immunity, and adaptive immunity. The results of KEGG pathway analysis indicated that DEGs in the high-risk and low-risk groups tended to be enriched in many pathways such as cholesterol metabolism, PPAR signaling pathway, complement and coagulation cascades, as well as the PI3K-Akt signaling pathway.

It is well known that lncRNAs are involved in the tumor immune microenvironment of tumors. They have been proved to play a significant role in various types of cancer [29, 30]. We examined the association between tumor-infiltrating immune cells and risk scores in the present study and determined that risk scores were adversely associated with the immune function and immune checkpoints. Thus, our study is the pioneer to investigate the correlations between cuproptosis-associated lncRNAs and tumor immunity in STSs. In our study, the high-risk group had significantly fewer tumor-infiltrating B cells, plasma cells, T cells, monocytes, and M1macrophages than the low-risk group. However, a higher proportion of cancer-infiltrating NK cells resting, as well as more M0 macrophages were found in high-risk patients. Studies have found that better outcomes are associated with many types of cells such as natural killer cells, tumor-infiltrating B cells, tumor-associated neutrophils (TANs), as well as dendritic cells. Conversely, the presence of tumor-associated macrophages (TAMs) was detrimental to the outcome [31]. These results were mostly consistent with our patients between high-risk and low-risk groups. As key and well-known regulatory immune checkpoint molecules, programmed death-1 (PD-1) as well as its ligand PD-L1 checkpoint pathway, in addition to immune checkpoint genes, including CTLA-4 and LAG3 play important roles in maintaining the balance between immune tolerance and autoimmunity [32]. The clinical benefit of immune checkpoint inhibitors (ICIs) has been well documented for several solid tumor types, such as malignant melanoma, lung, renal, urothelial, and head and neck cancer [17, 33–35]. However, the clinical benefits of ICIs for STSs have been controversial and generally unsatisfactory. In our study, we compared the immune checkpoint molecules between the high-risk group and low-risk group, and detected that the low-risk group exhibited much higher levels of expression of PDCD1, PDCD1LG2, CTLA4, CD274, HAVCR2, and IDO1. Notably, in STS patients, most reported studies showed that patients with higher immune checkpoint molecules are less likely to be benefited from immunotherapy, which was consistent with our findings. For example, researchers have mainly studied immunohistochemistry to evaluate these immune checkpoints and have ascertained the appearance of PD-1 and PD-L1, as well as their relation to poor outcomes [36–39]. There have been studies of other immune checkpoints in several tumors, however, there is only a limited number of reports for STSs. The expression of T cell immunoreceptors with Ig and ITIM domains (TIGIT) has recently been assessed in STSs. Despite TIGIT expression not being related to survival, CD155, its dominant ligand, did show to be related to a worse OS in the TCGA [40]. Yi et al. [41] used immunohistochemistry to investigate the expression of LAG3 and found that it is overexpressed on TILs. They also found that the expression of LAG3 is associated with a poor prognosis. Sporadically, PD-1 therapies might also be more effective in patients whose immune checkpoints are active. According to a study published in 2020, STS patients who reacted to anti-PD-1 immunotherapy of pembrolizumab had PD-L1-expressing macrophages in greater numbers than those who did not react [42].

In conclusion, due to the heterogeneity of STSs, a “one size fits all” approach will probably be less likely to be successful. In addition, a comprehensive immune profile, in conjunction with an assessment of clinical characteristics would be crucial for predicting the response and survival of ICIs.

Numerous studies have shown that for patients with high TMB, due to their relatively high number of neoantigens, immunotherapy might be more effective [43–45]. Our analysis of the patients' mutation data proved that the TMB was higher in high-risk patients compared with low-risk patients, indicating that immunotherapy might be more beneficial to high-risk patients. We also found that the mutation rate was 74 (69.81%) of 106 in the high-risk groups and the top three mutated genes were TP53, ATRX, and TTN, while the mutation rate was 78 (60.47%) of 129 samples in the low-risk and the top tree mutated genes were TP53, ATRX, and RB1. Notably, significantly more ATRX and TTN mutations were detected in patients with high risk than in patients with low risk. However, a completely opposite result was observed for mutation levels in TP53. TIDE is a computational framework that models the two main mechanisms of tumor immune escape, which can be used to predict immunotherapy responses [46, 47]. High TIDE predicts nonresponders in patients with suppressive cells that inhibit T-cell infiltration. To better demonstrate the predictive power of the risk score for immunotherapy, we applied TIDE in our cohort. We were surprised to find that there was a negative correlation between the TIDE and risk scores, further suggesting that high-risk patients might react more actively to immunotherapy.

In summary, it is demonstrated that the cuproptosis-associated lncRNA signature can effectively predict the tumor immune microenvironment in STS patients, and high-risk patients are more likely to have immunosensitive tumors that react more readily to immunotherapy. Furthermore, we also discovered that although low-risk patients had a better prognosis, however, they tend to have immunologically insensitive tumors that are hard to be treated by immunotherapy.

## 5. Conclusion

We developed a prognostic signature that has shown to be independent, highly reliable, and may provide some insight into future studies investigating the mechanisms between lncRNA and cuproptosis. Meanwhile, this study may provide new perceptions for patients with soft tissue sarcoma seeking immunotherapy.

### 5.1. Limitations

It is important to note that the study has several limitations. The first is that all analyses were performed on a public database, therefore, to further improve the reliability of the prediction results, we need to perform more in vivo and in vitro experimental studies to validate the newly established risk score model. Second, we were temporarily unable to obtain information about the expression levels of other lncRNAs supporting soft tissue sarcoma, clinical characteristics of patients, overall survival, and follow-up.

## Data Availability

The original data are maintained by the corresponding author. Information pertaining to the datasets will be made available upon written request.

## Supplementary Materials

Supplementary Materials Supplementary 1: clinical date of our cohort.

## References

[1] Coley W. B. (1910). The treatment of inoperable sarcoma by bacterial toxins (the mixed toxins of the Streptococcus erysipelas and the Bacillus prodigiosus). *Proceedings of the Royal Society of Medicine*.

[2] Gitto S., Cuocolo R., Albano D. (2021). CT and MRI radiomics of bone and soft-tissue sarcomas: a systematic review of reproducibility and validation strategies. *Insights Imaging*.

[3] Tseng W. W., Somaiah N., Engleman E. G. (2014). Potential for immunotherapy in soft tissue sarcoma. *Human Vaccines & Immunotherapeutics*.

[4] Mercer T. R., Dinger M. E., Mattick J. S. (2009). Long non-coding RNAs: insights into functions. *Nature Reviews Genetics*.

[5] Zhang X., Gejman R., Mahta A. (2010). Maternally expressed gene 3, an imprinted noncoding RNA gene, is associated with meningioma pathogenesis and progression. *Cancer Research*.

[6] Han G., Guo Q., Ma N. (2021). LncRNA BCRT1 facilitates osteosarcoma progression via regulating miR-1303/FGF7 axis. *Aging (Albany NY)*.

[7] Xiong J., Wu L., Huang L. (2021). LncRNA FOXP4-AS1 promotes progression of ewing sarcoma and is associated with immune infiltrates. *Frontiers Oncology*.

[8] Ji P., Diederichs S., Wang W. (2003). MALAT-1, a novel noncoding RNA, and thymosin *β*4 predict metastasis and survival in early-stage non-small cell lung cancer. *Oncogene*.

[9] Zhang L., Xu H. G., Lu C. (2014). A novel long non-coding RNA T-ALL-R-LncR1 knockdown and Par-4 cooperate to induce cellular apoptosis in T-cell acute lymphoblastic leukemia cells. *Leukemia and Lymphoma*.

[10] Miyoshi N., Wagatsuma H., Wakana S. (2000). Identification of an imprinted gene, Meg3/Gtl2 and its human homologue MEG3, first mapped on mouse distal chromosome 12 and human chromosome 14q. *Genes to Cells*.

[11] Akasbi Y., Awada A., Arifi S., Mellas N., El Mesbahi O. (2012). Non-HIV Kaposi’s sarcoma: a review and therapeutic perspectives. *Bulletin du Cancer*.

[12] Liu Y., Chen Q., Zhu Y. (2021). Non-coding RNAs in necroptosis, pyroptosis and ferroptosis in cancer metastasis. *Cell Death & Disease*.

[13] Tsvetkov P., Coy S., Petrova B. (2022). Copper induces cell death by targeting lipoylated TCA cycle proteins. *Science*.

[14] Ge E. J., Bush A. I., Casini A. (2022). Connecting copper and cancer: from transition metal signalling to metalloplasia. *Nature Reviews Cancer*.

[15] Blockhuys S., Celauro E., Hildesjö C. (2017). Defining the human copper proteome and analysis of its expression variation in cancers. *Metallomics*.

[16] Wang Y., Zhang L., Zhou F. (2022). Cuproptosis: a new form of programmed cell death. *Cellular and Molecular Immunology*.

[17] Carlino M. S., Larkin J., Long G. V. (2021). Immune checkpoint inhibitors in melanoma. *The Lancet*.

[18] Newman A. M., Liu C. L., Green M. R. (2015). Robust enumeration of cell subsets from tissue expression profiles. *Nature Methods*.

[19] Klempner S. J., Fabrizio D., Bane S. (2020). Tumor mutational burden as a predictive biomarker for response to immune checkpoint inhibitors: a review of current evidence. *The Oncologist*.

[20] Jiang P., Gu S., Pan D. (2018). Signatures of T cell dysfunction and exclusion predict cancer immunotherapy response. *Nature Medicine*.

[21] Vrieze S. I. (2012). Model selection and psychological theory: a discussion of the differences between the Akaike information criterion (AIC) and the Bayesian information criterion (BIC). *Psychological Methods*.

[22] Saerens M., Brusselaers N., Rottey S., Decruyenaere A., Creytens D., Lapeire L. (2021). Immune checkpoint inhibitors in treatment of soft-tissue sarcoma: a systematic review and meta-analysis. *European Journal of Cancer*.

[23] Kask G., Repo J. P., Tukiainen E. J., Blomqvist C., Barner-Rasmussen I. (2021). Soft tissue sarcoma of lower extremity: functional outcome and quality of life. *Annals of Surgical Oncology*.

[24] Shah N. K., Yegya-Raman N., Jones J. A., Shabason J. E. (2021). Radiation therapy in metastatic soft tissue sarcoma: from palliation to ablation. *Cancers*.

[25] Liu S. J., Dang H. X., Lim D. A., Feng F. Y., Maher C. A. (2021). Long noncoding RNAs in cancer metastasis. *Nature Reviews Cancer*.

[26] Cai Q., Zhao X., Wang Y. (2021). LINC01614 promotes osteosarcoma progression via miR-520a-3p/SNX3 axis. *Cellular Signalling*.

[27] Li H., Huang F., Liu X. Q., Liu H. C., Dai M., Zeng J. (2021). LncRNA TUG1 promotes Ewing’s sarcoma cell proliferation, migration, and invasion via the miR-199a-3p-MSI2 signaling pathway. *Neoplasma*.

[28] Bae J. Y., Choi K. U., Kim A. (2020). Evaluation of immune-biomarker expression in high-grade soft-tissue sarcoma: HLA-DQA1 expression as a prognostic marker. *Experimental and Therapeutic Medicine*.

[29] Huang Q., Lin Y., Chen C. (2021). Immune-related LncRNAs affect the prognosis of osteosarcoma, which are related to the tumor immune microenvironment. *Frontiers in Cell and Developmental Biology*.

[30] Qin M., Ma Y., Wang Z., Fang D., Wei J. (2021). Using immune-related lncRNAs to construct novel biomarkers and investigate the immune landscape of breast cancer. *Translational Cancer Research*.

[31] Sousa L. M., Almeida J. S., Fortes-Andrade T. (2021). Tumor and peripheral immune status in soft tissue sarcoma: implications for immunotherapy. *Cancers*.

[32] Keir M. E., Butte M. J., Freeman G. J., Sharpe A. H. (2008). PD-1 and its ligands in tolerance and immunity. *Annual Review of Immunology*.

[33] Bedke J., Albiges L., Capitanio U. (2021). The 2021 updated European association of urology guidelines on renal cell carcinoma: immune checkpoint inhibitor-based combination therapies for treatment-naive metastatic clear-cell renal cell carcinoma are standard of care. *European Urology*.

[34] Morad G., Helmink B. A., Sharma P., Wargo J. A. (2021). Hallmarks of response, resistance, and toxicity to immune checkpoint blockade. *Cell*.

[35] Ferrarotto R., Amit M., Nagarajan P. (2021). Pilot phase II trial of neoadjuvant immunotherapy in locoregionally advanced, resectable cutaneous squamous cell carcinoma of the head and neck. *Clinical Cancer Research*.

[36] Torabi A., Amaya C. N., Wians F. H., Bryan B. A. (2017). PD-1 and PD-L1 expression in bone and soft tissue sarcomas. *Pathology*.

[37] Orth M. F., Buecklein V. L., Kampmann E. (2020). A comparative view on the expression patterns of PD-L1 and PD-1 in soft tissue sarcomas. *Cancer Immunology Immunotherapy*.

[38] Kim C., Kim E. K., Jung H. (2016). Prognostic implications of PD-L1 expression in patients with soft tissue sarcoma. *BMC Cancer*.

[39] Paydas S., Bagir E. K., Deveci M. A., Gonlusen G. (2016). Clinical and prognostic significance of PD-1 and PD-L1 expression in sarcomas. *Medical Oncology*.

[40] Judge S. J., Darrow M. A., Thorpe S. W. (2020). Analysis of tumor-infiltrating NK and T cells highlights IL-15 stimulation and TIGIT blockade as a combination immunotherapy strategy for soft tissue sarcomas. *Journal for Immunotherapy Cancer*.

[41] Yi Q., Zhixin F., Yuanxiang G. (2019). LAG-3 expression on tumor-infiltrating T cells in soft tissue sarcoma correlates with poor survival. *Cancer Biology & Medicine*.

[42] Keung E. Z., Burgess M., Salazar R. (2020). Correlative analyses of the SARC028 trial reveal an association between sarcoma-associated immune infiltrate and response to pembrolizumab. *Clinical Cancer Research*.

[43] Zheng Y., Yao M., Yang Y. (2021). Higher tumor mutation burden was a predictor for better outcome for nsclc patients treated with PD-1 antibodies: a systematic review and meta-analysis. *SLAS Technology*.

[44] Li Y., Ma Y., Wu Z. (2021). Tumor mutational burden predicting the efficacy of immune checkpoint inhibitors in colorectal cancer: a systematic review and meta-analysis. *Frontiers in Immunology*.

[45] Deng H., Zhao Y., Cai X. (2022). PD-L1 expression and Tumor mutation burden as Pathological response biomarkers of Neoadjuvant immunotherapy for Early-stage Non-small cell lung cancer: a systematic review and meta-analysis. *Critical Reviews in Oncology*.

[46] Wu J., Li L., Zhang H. (2021). A risk model developed based on tumor microenvironment predicts overall survival and associates with tumor immunity of patients with lung adenocarcinoma. *Oncogene*.

[47] Chen X., Xu R., He D. (2021). CD8 (+) T effector and immune checkpoint signatures predict prognosis and responsiveness to immunotherapy in bladder cancer. *Oncogene*.

