# Supplementary material for: A Newly Established Cuproptosis-Associated Long Non-Coding RNA Signature for Predicting Prognosis and Indicating Immune Microenvironment Features in Soft Tissue Sarcoma
Source: J Oncol. 2022 Jul 6;2022:8489387. doi: 10.1155/2022/8489387 (PMC9279026; doi:10.1155/2022/8489387)
Supplement: Supplementary Materials — Supplementary 1: clinical date of our cohort. [file 8489387.f1.pdf]

Id	futime	fustat	Age	Gender	Grade	Stage	T	M	N
TCGA-K1-	1622	0	unknow	MALE	unknow	unknow	unknow	unknow	unknow
TCGA-X6-	628	1	unknow	FEMALE	unknow	unknow	unknow	unknow	unknow
TCGA-DX	610	0	unknow	FEMALE	unknow	unknow	unknow	unknow	unknow
TCGA-DX	427	1	unknow	MALE	unknow	unknow	unknow	unknow	unknow
TCGA-3B-	1521	0	unknow	MALE	unknow	unknow	unknow	unknow	unknow
TCGA-DX	2036	0	unknow	MALE	unknow	unknow	unknow	unknow	unknow
TCGA-X6-	938	0	unknow	FEMALE	unknow	unknow	unknow	unknow	unknow
TCGA-DX	1073	0	unknow	MALE	unknow	unknow	unknow	unknow	unknow
TCGA-3B-	1665	0	unknow	MALE	unknow	unknow	unknow	unknow	unknow
TCGA-QC	398	0	unknow	MALE	unknow	unknow	unknow	unknow	unknow
TCGA-3B-	959	0	unknow	MALE	unknow	unknow	unknow	unknow	unknow
TCGA-LI-	922	0	unknow	FEMALE	unknow	unknow	unknow	unknow	unknow
TCGA-PC-	1175	1	unknow	FEMALE	unknow	unknow	unknow	unknow	unknow
TCGA-DX	1373	0	unknow	MALE	unknow	unknow	unknow	unknow	unknow
TCGA-PC-	550	1	unknow	FEMALE	unknow	unknow	unknow	unknow	unknow
TCGA-IS-	4845	0	unknow	FEMALE	unknow	unknow	unknow	unknow	unknow
TCGA-QC	550	1	unknow	FEMALE	unknow	unknow	unknow	unknow	unknow
TCGA-DX	738	1	unknow	FEMALE	unknow	unknow	unknow	unknow	unknow
TCGA-DX	897	1	unknow	MALE	unknow	unknow	unknow	unknow	unknow
TCGA-DX	295	1	unknow	MALE	unknow	unknow	unknow	unknow	unknow
TCGA-DX	200	1	unknow	FEMALE	unknow	unknow	unknow	unknow	unknow
TCGA-JV-	499	0	unknow	FEMALE	unknow	unknow	unknow	unknow	unknow
TCGA-DX	3156	0	unknow	MALE	unknow	unknow	unknow	unknow	unknow
TCGA-DX	284	1	unknow	MALE	unknow	unknow	unknow	unknow	unknow
TCGA-FX-	471	0	unknow	FEMALE	unknow	unknow	unknow	unknow	unknow
TCGA-DX	296	1	unknow	MALE	unknow	unknow	unknow	unknow	unknow
TCGA-DX	513	1	unknow	FEMALE	unknow	unknow	unknow	unknow	unknow
TCGA-IE-	819	0	unknow	FEMALE	unknow	unknow	unknow	unknow	unknow
TCGA-DX	2599	1	unknow	FEMALE	unknow	unknow	unknow	unknow	unknow
TCGA-3B-	314	0	unknow	MALE	unknow	unknow	unknow	unknow	unknow
TCGA-DX	2324	1	unknow	MALE	unknow	unknow	unknow	unknow	unknow
TCGA-DX	2034	1	unknow	FEMALE	unknow	unknow	unknow	unknow	unknow
TCGA-K1-	1891	0	unknow	FEMALE	unknow	unknow	unknow	unknow	unknow
TCGA-FX-	618	0	unknow	MALE	unknow	unknow	unknow	unknow	unknow
TCGA-SI-	771	0	unknow	MALE	unknow	unknow	unknow	unknow	unknow
TCGA-IW-	180	1	unknow	FEMALE	unknow	unknow	unknow	unknow	unknow
TCGA-DX	1240	0	unknow	FEMALE	unknow	unknow	unknow	unknow	unknow
TCGA-KD	1073	1	unknow	FEMALE	unknow	unknow	unknow	unknow	unknow
TCGA-X9-	1108	0	unknow	MALE	unknow	unknow	unknow	unknow	unknow
TCGA-HS	22	1	unknow	FEMALE	unknow	unknow	unknow	unknow	unknow
TCGA-DX	138	1	unknow	MALE	unknow	unknow	unknow	unknow	unknow
TCGA-DX	365	0	unknow	FEMALE	unknow	unknow	unknow	unknow	unknow
TCGA-IE-	594	0	unknow	FEMALE	unknow	unknow	unknow	unknow	unknow
TCGA-IS-	413	1	unknow	FEMALE	unknow	unknow	unknow	unknow	unknow
TCGA-X6-	1067	1	unknow	MALE	unknow	unknow	unknow	unknow	unknow
TCGA-DX	1552	1	unknow	MALE	unknow	unknow	unknow	unknow	unknow
TCGA-K1-	3765	0	unknow	FEMALE	unknow	unknow	unknow	unknow	unknow
TCGA-VT-	379	0	unknow	MALE	unknow	unknow	unknow	unknow	unknow
TCGA-DX	1536	1	unknow	MALE	unknow	unknow	unknow	unknow	unknow
TCGA-DX	1055	0	unknow	MALE	unknow	unknow	unknow	unknow	unknow
TCGA-DX	280	0	unknow	FEMALE	unknow	unknow	unknow	unknow	unknow
TCGA-DX	1548	0	unknow	FEMALE	unknow	unknow	unknow	unknow	unknow
TCGA-DX	2085	0	unknow	FEMALE	unknow	unknow	unknow	unknow	unknow
TCGA-DX	1230	0	unknow	FEMALE	unknow	unknow	unknow	unknow	unknow
TCGA-DX	1400	0	unknow	FEMALE	unknow	unknow	unknow	unknow	unknow
TCGA-MC	318	1	unknow	FEMALE	unknow	unknow	unknow	unknow	unknow
TCGA-DX	2381	0	unknow	MALE	unknow	unknow	unknow	unknow	unknow
TCGA-IS-	858	1	unknow	FEMALE	unknow	unknow	unknow	unknow	unknow

TCGA-DX	1399	0	unknow	MALE	unknow	unknow	unknow	unknow	unknow
TCGA-IW-	688	0	unknow	FEMALE	unknow	unknow	unknow	unknow	unknow
TCGA-HS-	1585	0	unknow	FEMALE	unknow	unknow	unknow	unknow	unknow
TCGA-DX	191	1	unknow	MALE	unknow	unknow	unknow	unknow	unknow
TCGA-MB	773	0	unknow	FEMALE	unknow	unknow	unknow	unknow	unknow
TCGA-HB-	384	1	unknow	FEMALE	unknow	unknow	unknow	unknow	unknow
TCGA-DX	969	0	unknow	MALE	unknow	unknow	unknow	unknow	unknow
TCGA-VT-	796	0	unknow	FEMALE	unknow	unknow	unknow	unknow	unknow
TCGA-SI-/	694	1	unknow	MALE	unknow	unknow	unknow	unknow	unknow
TCGA-FX-	505	0	unknow	MALE	unknow	unknow	unknow	unknow	unknow
TCGA-FX-	1495	0	unknow	FEMALE	unknow	unknow	unknow	unknow	unknow
TCGA-DX	1359	0	unknow	MALE	unknow	unknow	unknow	unknow	unknow
TCGA-QQ	3406	0	unknow	MALE	unknow	unknow	unknow	unknow	unknow
TCGA-DX	1092	0	unknow	FEMALE	unknow	unknow	unknow	unknow	unknow
TCGA-DX	2044	0	unknow	FEMALE	unknow	unknow	unknow	unknow	unknow
TCGA-IE-/	2448	1	unknow	MALE	unknow	unknow	unknow	unknow	unknow
TCGA-WK	146	1	unknow	MALE	unknow	unknow	unknow	unknow	unknow
TCGA-DX	1020	1	unknow	FEMALE	unknow	unknow	unknow	unknow	unknow
TCGA-FX-	245	1	unknow	FEMALE	unknow	unknow	unknow	unknow	unknow
TCGA-DX	485	1	unknow	MALE	unknow	unknow	unknow	unknow	unknow
TCGA-DX	928	0	unknow	MALE	unknow	unknow	unknow	unknow	unknow
TCGA-DX	325	1	unknow	FEMALE	unknow	unknow	unknow	unknow	unknow
TCGA-WP	261	1	unknow	FEMALE	unknow	unknow	unknow	unknow	unknow
TCGA-DX	1579	0	unknow	MALE	unknow	unknow	unknow	unknow	unknow
TCGA-DX	4573	0	unknow	FEMALE	unknow	unknow	unknow	unknow	unknow
TCGA-DX	1105	0	unknow	MALE	unknow	unknow	unknow	unknow	unknow
TCGA-WK	363	1	unknow	FEMALE	unknow	unknow	unknow	unknow	unknow
TCGA-3B-	808	0	unknow	MALE	unknow	unknow	unknow	unknow	unknow
TCGA-DX	2408	0	unknow	MALE	unknow	unknow	unknow	unknow	unknow
TCGA-MJ-	456	0	unknow	FEMALE	unknow	unknow	unknow	unknow	unknow
TCGA-DX	1534	0	unknow	MALE	unknow	unknow	unknow	unknow	unknow
TCGA-DX	2007	0	unknow	MALE	unknow	unknow	unknow	unknow	unknow
TCGA-DX	3238	0	unknow	FEMALE	unknow	unknow	unknow	unknow	unknow
TCGA-IE-/	42	0	unknow	MALE	unknow	unknow	unknow	unknow	unknow
TCGA-DX	1247	0	unknow	MALE	unknow	unknow	unknow	unknow	unknow
TCGA-DX	1372	0	unknow	MALE	unknow	unknow	unknow	unknow	unknow
TCGA-DX	2619	0	unknow	FEMALE	unknow	unknow	unknow	unknow	unknow
TCGA-QC	537	0	unknow	FEMALE	unknow	unknow	unknow	unknow	unknow
TCGA-FX-	605	1	unknow	FEMALE	unknow	unknow	unknow	unknow	unknow
TCGA-X6-	583	0	unknow	FEMALE	unknow	unknow	unknow	unknow	unknow
TCGA-DX	93	1	unknow	FEMALE	unknow	unknow	unknow	unknow	unknow
TCGA-DX	463	0	unknow	MALE	unknow	unknow	unknow	unknow	unknow
TCGA-DX	2247	0	unknow	MALE	unknow	unknow	unknow	unknow	unknow
TCGA-QQ	1478	1	unknow	FEMALE	unknow	unknow	unknow	unknow	unknow
TCGA-DX	275	1	unknow	FEMALE	unknow	unknow	unknow	unknow	unknow
TCGA-N1-	224	1	unknow	FEMALE	unknow	unknow	unknow	unknow	unknow
TCGA-DX	456	1	unknow	MALE	unknow	unknow	unknow	unknow	unknow
TCGA-DX	1885	0	unknow	FEMALE	unknow	unknow	unknow	unknow	unknow
TCGA-DX	995	1	unknow	MALE	unknow	unknow	unknow	unknow	unknow
TCGA-WK	1722	1	unknow	FEMALE	unknow	unknow	unknow	unknow	unknow
TCGA-3B-	2694	1	unknow	FEMALE	unknow	unknow	unknow	unknow	unknow
TCGA-WK	1164	1	unknow	MALE	unknow	unknow	unknow	unknow	unknow
TCGA-X6-	0	0	unknow	FEMALE	unknow	unknow	unknow	unknow	unknow
TCGA-3B-	1096	0	unknow	MALE	unknow	unknow	unknow	unknow	unknow
TCGA-MJ-	681	0	unknow	MALE	unknow	unknow	unknow	unknow	unknow
TCGA-WK	2579	0	unknow	FEMALE	unknow	unknow	unknow	unknow	unknow
TCGA-DX	1116	1	unknow	MALE	unknow	unknow	unknow	unknow	unknow
TCGA-IF-/	767	1	unknow	MALE	unknow	unknow	unknow	unknow	unknow
TCGA-3R-	17	1	unknow	MALE	unknow	unknow	unknow	unknow	unknow

TCGA-3B-	1366	1	unknow	MALE	unknow	unknow	unknow	unknow	unknow
TCGA-SG-	576	0	unknow	MALE	unknow	unknow	unknow	unknow	unknow
TCGA-X6-	1070	0	unknow	MALE	unknow	unknow	unknow	unknow	unknow
TCGA-X6-	24	1	unknow	MALE	unknow	unknow	unknow	unknow	unknow
TCGA-QQ	3964	0	unknow	MALE	unknow	unknow	unknow	unknow	unknow
TCGA-DX	1456	0	unknow	MALE	unknow	unknow	unknow	unknow	unknow
TCGA-DX	2830	0	unknow	FEMALE	unknow	unknow	unknow	unknow	unknow
TCGA-QQ	1092	0	unknow	FEMALE	unknow	unknow	unknow	unknow	unknow
TCGA-DX	160	1	unknow	FEMALE	unknow	unknow	unknow	unknow	unknow
TCGA-FX-	660	0	unknow	FEMALE	unknow	unknow	unknow	unknow	unknow
TCGA-DX	1088	1	unknow	FEMALE	unknow	unknow	unknow	unknow	unknow
TCGA-DX	35	1	unknow	MALE	unknow	unknow	unknow	unknow	unknow
TCGA-QQ	1129	0	unknow	MALE	unknow	unknow	unknow	unknow	unknow
TCGA-DX	821	0	unknow	MALE	unknow	unknow	unknow	unknow	unknow
TCGA-DX	3310	0	unknow	MALE	unknow	unknow	unknow	unknow	unknow
TCGA-UE-	0	0	unknow	FEMALE	unknow	unknow	unknow	unknow	unknow
TCGA-DX	1492	0	unknow	MALE	unknow	unknow	unknow	unknow	unknow
TCGA-DX	1143	0	unknow	MALE	unknow	unknow	unknow	unknow	unknow
TCGA-DX	564	1	unknow	MALE	unknow	unknow	unknow	unknow	unknow
TCGA-VT-	326	0	unknow	MALE	unknow	unknow	unknow	unknow	unknow
TCGA-DX	695	1	unknow	FEMALE	unknow	unknow	unknow	unknow	unknow
TCGA-MC	352	0	unknow	FEMALE	unknow	unknow	unknow	unknow	unknow
TCGA-DX	482	0	unknow	FEMALE	unknow	unknow	unknow	unknow	unknow
TCGA-HB-	139	1	unknow	MALE	unknow	unknow	unknow	unknow	unknow
TCGA-MB	15	0	unknow	MALE	unknow	unknow	unknow	unknow	unknow
TCGA-QQ	5723	0	unknow	FEMALE	unknow	unknow	unknow	unknow	unknow
TCGA-X9-	831	0	unknow	FEMALE	unknow	unknow	unknow	unknow	unknow
TCGA-DX	1649	1	unknow	MALE	unknow	unknow	unknow	unknow	unknow
TCGA-X6-	550	1	unknow	FEMALE	unknow	unknow	unknow	unknow	unknow
TCGA-DX	4627	0	unknow	FEMALE	unknow	unknow	unknow	unknow	unknow
TCGA-WK	688	1	unknow	FEMALE	unknow	unknow	unknow	unknow	unknow
TCGA-HS-	1013	0	unknow	FEMALE	unknow	unknow	unknow	unknow	unknow
TCGA-PT-	813	0	unknow	FEMALE	unknow	unknow	unknow	unknow	unknow
TCGA-K1-	711	1	unknow	FEMALE	unknow	unknow	unknow	unknow	unknow
TCGA-DX	570	1	unknow	FEMALE	unknow	unknow	unknow	unknow	unknow
TCGA-RN	22	0	unknow	FEMALE	unknow	unknow	unknow	unknow	unknow
TCGA-SG-	118	1	unknow	FEMALE	unknow	unknow	unknow	unknow	unknow
TCGA-MJ-	547	0	unknow	FEMALE	unknow	unknow	unknow	unknow	unknow
TCGA-DX	2515	0	unknow	FEMALE	unknow	unknow	unknow	unknow	unknow
TCGA-MB	0	0	unknow	MALE	unknow	unknow	unknow	unknow	unknow
TCGA-DX	439	1	unknow	MALE	unknow	unknow	unknow	unknow	unknow
TCGA-DX	2901	0	unknow	MALE	unknow	unknow	unknow	unknow	unknow
TCGA-DX	850	1	unknow	FEMALE	unknow	unknow	unknow	unknow	unknow
TCGA-FX-	947	0	unknow	MALE	unknow	unknow	unknow	unknow	unknow
TCGA-IE-A	98	0	unknow	FEMALE	unknow	unknow	unknow	unknow	unknow
TCGA-JV-	119	1	unknow	FEMALE	unknow	unknow	unknow	unknow	unknow
TCGA-MB	661	0	unknow	MALE	unknow	unknow	unknow	unknow	unknow
TCGA-HB-	2057	0	unknow	FEMALE	unknow	unknow	unknow	unknow	unknow
TCGA-JV-	32	1	unknow	FEMALE	unknow	unknow	unknow	unknow	unknow
TCGA-DX	1834	0	unknow	MALE	unknow	unknow	unknow	unknow	unknow
TCGA-DX	1820	0	unknow	MALE	unknow	unknow	unknow	unknow	unknow
TCGA-DX	541	0	unknow	MALE	unknow	unknow	unknow	unknow	unknow
TCGA-QQ	1970	1	unknow	MALE	unknow	unknow	unknow	unknow	unknow
TCGA-IW-	2615	0	unknow	FEMALE	unknow	unknow	unknow	unknow	unknow
TCGA-X6-	896	0	unknow	FEMALE	unknow	unknow	unknow	unknow	unknow
TCGA-DX	972	0	unknow	FEMALE	unknow	unknow	unknow	unknow	unknow
TCGA-PC-	3740	0	unknow	MALE	unknow	unknow	unknow	unknow	unknow
TCGA-HB-	1126	0	unknow	FEMALE	unknow	unknow	unknow	unknow	unknow
TCGA-DX	2087	0	unknow	FEMALE	unknow	unknow	unknow	unknow	unknow

TCGA-K1-	3080	0	unknow	FEMALE	unknow	unknow	unknow	unknow	unknow
TCGA-DX	2271	0	unknow	FEMALE	unknow	unknow	unknow	unknow	unknow
TCGA-DX	846	1	unknow	MALE	unknow	unknow	unknow	unknow	unknow
TCGA-DX	2291	0	unknow	FEMALE	unknow	unknow	unknow	unknow	unknow
TCGA-DX	1121	0	unknow	MALE	unknow	unknow	unknow	unknow	unknow
TCGA-MB	600	0	unknow	FEMALE	unknow	unknow	unknow	unknow	unknow
TCGA-DX	1168	1	unknow	MALE	unknow	unknow	unknow	unknow	unknow
TCGA-IF-	1590	0	unknow	FEMALE	unknow	unknow	unknow	unknow	unknow
TCGA-3B-	599	1	unknow	MALE	unknow	unknow	unknow	unknow	unknow
TCGA-DX	1466	1	unknow	MALE	unknow	unknow	unknow	unknow	unknow
TCGA-HS-	298	0	unknow	FEMALE	unknow	unknow	unknow	unknow	unknow
TCGA-DX	1845	1	unknow	MALE	unknow	unknow	unknow	unknow	unknow
TCGA-3B-	768	0	unknow	FEMALE	unknow	unknow	unknow	unknow	unknow
TCGA-X6-	1547	0	unknow	FEMALE	unknow	unknow	unknow	unknow	unknow
TCGA-DX	923	0	unknow	FEMALE	unknow	unknow	unknow	unknow	unknow
TCGA-WK	1991	1	unknow	FEMALE	unknow	unknow	unknow	unknow	unknow
TCGA-DX	591	1	unknow	MALE	unknow	unknow	unknow	unknow	unknow
TCGA-Z4-	485	0	unknow	FEMALE	unknow	unknow	unknow	unknow	unknow
TCGA-X2-	1927	0	unknow	FEMALE	unknow	unknow	unknow	unknow	unknow
TCGA-DX	2575	1	unknow	FEMALE	unknow	unknow	unknow	unknow	unknow
TCGA-PC-	2464	1	unknow	FEMALE	unknow	unknow	unknow	unknow	unknow
TCGA-PC-	193	1	unknow	MALE	unknow	unknow	unknow	unknow	unknow
TCGA-QC	56	0	unknow	MALE	unknow	unknow	unknow	unknow	unknow
TCGA-SI-	262	1	unknow	FEMALE	unknow	unknow	unknow	unknow	unknow
TCGA-DX	1161	0	unknow	FEMALE	unknow	unknow	unknow	unknow	unknow
TCGA-DX	721	0	unknow	FEMALE	unknow	unknow	unknow	unknow	unknow
TCGA-DX	1045	0	unknow	FEMALE	unknow	unknow	unknow	unknow	unknow
TCGA-DX	3102	0	unknow	MALE	unknow	unknow	unknow	unknow	unknow
TCGA-DX	492	1	unknow	FEMALE	unknow	unknow	unknow	unknow	unknow
TCGA-WK	1262	1	unknow	FEMALE	unknow	unknow	unknow	unknow	unknow
TCGA-QC	320	1	unknow	FEMALE	unknow	unknow	unknow	unknow	unknow
TCGA-SI-	996	0	unknow	FEMALE	unknow	unknow	unknow	unknow	unknow
TCGA-UE-	402	0	unknow	FEMALE	unknow	unknow	unknow	unknow	unknow
TCGA-DX	1138	0	unknow	FEMALE	unknow	unknow	unknow	unknow	unknow
TCGA-DX	2030	0	unknow	FEMALE	unknow	unknow	unknow	unknow	unknow
TCGA-IF-	1129	1	unknow	FEMALE	unknow	unknow	unknow	unknow	unknow
TCGA-3B-	567	1	unknow	FEMALE	unknow	unknow	unknow	unknow	unknow
TCGA-DX	2169	0	unknow	MALE	unknow	unknow	unknow	unknow	unknow
TCGA-QC	1424	1	unknow	MALE	unknow	unknow	unknow	unknow	unknow
TCGA-KD-	1941	1	unknow	FEMALE	unknow	unknow	unknow	unknow	unknow
TCGA-DX	391	1	unknow	MALE	unknow	unknow	unknow	unknow	unknow
TCGA-DX	2586	0	unknow	MALE	unknow	unknow	unknow	unknow	unknow
TCGA-DX	478	0	unknow	MALE	unknow	unknow	unknow	unknow	unknow
TCGA-LI-	540	1	unknow	FEMALE	unknow	unknow	unknow	unknow	unknow
TCGA-DX	1892	0	unknow	MALE	unknow	unknow	unknow	unknow	unknow
TCGA-DX	1235	1	unknow	FEMALE	unknow	unknow	unknow	unknow	unknow
TCGA-3B-	1104	0	unknow	MALE	unknow	unknow	unknow	unknow	unknow
TCGA-DX	881	0	unknow	MALE	unknow	unknow	unknow	unknow	unknow
TCGA-DX	404	1	unknow	FEMALE	unknow	unknow	unknow	unknow	unknow
TCGA-Z4-	407	0	unknow	MALE	unknow	unknow	unknow	unknow	unknow
TCGA-DX	261	0	unknow	MALE	unknow	unknow	unknow	unknow	unknow
TCGA-DX	805	0	unknow	MALE	unknow	unknow	unknow	unknow	unknow
TCGA-SI-	597	0	unknow	FEMALE	unknow	unknow	unknow	unknow	unknow
TCGA-KD-	499	0	unknow	FEMALE	unknow	unknow	unknow	unknow	unknow
TCGA-SG-	533	0	unknow	MALE	unknow	unknow	unknow	unknow	unknow
TCGA-DX	1953	1	unknow	FEMALE	unknow	unknow	unknow	unknow	unknow
TCGA-IE-	605	0	unknow	FEMALE	unknow	unknow	unknow	unknow	unknow
TCGA-DX	367	1	unknow	MALE	unknow	unknow	unknow	unknow	unknow
TCGA-IS-	5204	0	unknow	FEMALE	unknow	unknow	unknow	unknow	unknow

TCGA-DX	1049	1	unknow	FEMALE	unknow	unknow	unknow	unknow	unknow
TCGA-3B-	1627	1	unknow	FEMALE	unknow	unknow	unknow	unknow	unknow
TCGA-K1-	158	0	unknow	MALE	unknow	unknow	unknow	unknow	unknow
TCGA-3B-	437	1	unknow	FEMALE	unknow	unknow	unknow	unknow	unknow
TCGA-RN	510	0	unknow	MALE	unknow	unknow	unknow	unknow	unknow
TCGA-DX	428	0	unknow	MALE	unknow	unknow	unknow	unknow	unknow
TCGA-DX	4150	0	unknow	MALE	unknow	unknow	unknow	unknow	unknow
TCGA-DX	978	1	unknow	MALE	unknow	unknow	unknow	unknow	unknow
TCGA-DX	1061	1	unknow	FEMALE	unknow	unknow	unknow	unknow	unknow
TCGA-KF-	356	0	unknow	FEMALE	unknow	unknow	unknow	unknow	unknow
TCGA-DX	1605	0	unknow	FEMALE	unknow	unknow	unknow	unknow	unknow
TCGA-3B-	1303	0	unknow	FEMALE	unknow	unknow	unknow	unknow	unknow
TCGA-K1-	528	0	unknow	MALE	unknow	unknow	unknow	unknow	unknow
TCGA-X6-	1379	0	unknow	MALE	unknow	unknow	unknow	unknow	unknow
TCGA-DX	2641	0	unknow	MALE	unknow	unknow	unknow	unknow	unknow
TCGA-DX	1017	0	unknow	MALE	unknow	unknow	unknow	unknow	unknow
TCGA-HB-	1061	1	unknow	FEMALE	unknow	unknow	unknow	unknow	unknow
TCGA-DX	546	0	unknow	FEMALE	unknow	unknow	unknow	unknow	unknow
TCGA-QC	637	0	unknow	MALE	unknow	unknow	unknow	unknow	unknow
TCGA-DX	2625	0	unknow	MALE	unknow	unknow	unknow	unknow	unknow
TCGA-PC-	1142	1	unknow	MALE	unknow	unknow	unknow	unknow	unknow
TCGA-3B-	1170	0	unknow	MALE	unknow	unknow	unknow	unknow	unknow
TCGA-3B-	2085	0	unknow	FEMALE	unknow	unknow	unknow	unknow	unknow
TCGA-Z4-	133	0	unknow	FEMALE	unknow	unknow	unknow	unknow	unknow
TCGA-DX	2369	0	unknow	FEMALE	unknow	unknow	unknow	unknow	unknow
TCGA-Z4-	486	0	unknow	FEMALE	unknow	unknow	unknow	unknow	unknow
